# Overcoming public resistance to carbon taxes

**DOI:** 10.1002/wcc.531

**Published:** 2018-06-06

**Authors:** Stefano Carattini, Maria Carvalho, Sam Fankhauser

**Affiliations:** ^1^ Yale School of Forestry & Environmental Studies Yale University New Haven Connecticut; ^2^ Grantham Research Institute and CCCEP London School of Economics and Political Science London UK

**Keywords:** acceptability, carbon pricing, carbon taxes, public support, revenue recycling

## Abstract

Carbon taxes represent a cost‐effective way to steer the economy toward a greener future. In the real world, their application has however been limited. In this paper, we address one of the main obstacles to carbon taxes: public opposition. We identify drivers of and barriers to public support, and, under the form of stylized facts, provide general lessons on the acceptability of carbon taxes. We derive our lessons from a growing literature, as well as from a combination of policy “failures” and “successes.” Based on our stylized facts, we formulate a set of suggestions concerning the design of carbon taxes. We consider the use of trial periods, tax escalators, environmental earmarking, lump‐sum transfers, tax rebates, and advanced communication strategies, among others. This paper contributes to the policy debate about carbon taxes, hopefully leading to more success stories and fewer policy failures.

This article is categorized under:

Climate Economics > Economics of Mitigation

## 1 INTRODUCTION

Putting a price on carbon is central to effective climate policy. Global efforts to reduce greenhouse gas emissions need to step up in all economic sectors to meet the Paris Agreement target: to keep the rise in global mean temperatures well below 2 °C above preindustrial levels. This requires a variety of policy interventions, including subsidies to support the breakthrough of low‐carbon technologies, regulatory standards to drive down the energy use of buildings, cars and appliances, and financing schemes to overcome capital constraints (Bowen & Fankhauser, 2017). However, an effective carbon price is essential to avoid more severe interferences with the climate system (Stiglitz et al., 2018). Only if the emitters of greenhouse gases face the full environmental costs of their actions will they manage their carbon emissions effectively. Carbon pricing alters relative prices, leading to an automatic adjustment in behavior by firms and consumers, and creating a continuous incentive for investments in low‐carbon technological improvements. It works as a decentralized policy, in that it does not require regulators to have information on marginal abatement costs. Agents react to the carbon price based on their marginal abatement cost. By exploiting heterogeneity in marginal abatement costs, carbon pricing allows reducing the overall abatement cost (Weitzman, 1974).

Until now, emissions trading has been the carbon pricing instrument of choice in most jurisdictions. In the European Union, the EU Emissions Trading System (EU ETS) covers almost half of total greenhouse gas emissions. Carbon is also traded in Canada, China, New Zealand, Switzerland, and the United States, although most of these schemes are limited in their regional or sectoral scope (World Bank, 2016).

Carbon taxation, in conjunction with other regulatory measures, could be an effective way of closing policy gaps in sectors that are not already covered by a functioning emissions trading system. In the EU, carbon taxes could play a role in reducing emissions outside the EU ETS, where much of the future policy effort must lie, according to the European Environment Agency (2016). Taxation may also play a larger role in the United Kingdom as it seeks to meet its carbon targets after Brexit. The German Renewable Energy Federation has advocated for replacing the existing power tax with a national carbon tax for electricity, thereby providing an alternative financing solution to expanding renewable capacity as part of Germany's low‐carbon energy transition (Wehrmann, 2017). In the United States, senior Republicans have laid out their arguments for a US$40 carbon tax in *The Conservative Case for Carbon Dividends* (Baker III, Feldstein, Halstead, et al., 2017).

A carbon tax is a relatively simple instrument to impose on the individual emitters, including the many smaller ones that dominate the non‐ETS sectors and are less likely than large emitting facilities or sources to engage in carbon trading. According to the expertise collected by the World Bank, cap‐and‐trade systems—like the EU ETS—are best suited for industrial actors that have the capacity and skills to engage in the market actively (World Bank, 2016). With their high transaction costs, such systems are less appealing for sectors with a large number of small emission sources, such as transportation and buildings (Goulder & Parry, 2008). Economists advocate the use of carbon taxes because they provide the price incentive to reduce emissions without being technologically prescriptive, are simpler to administer, and do not draw on government budgets (Aldy & Stavins, 2012; Baranzini et al., 2017; Baumol & Oates, 1971; Goulder & Parry, 2008; Mankiw, 2009; Metcalf, 2009; Weitzman, 2015).

Despite these advantages, carbon taxes are one of the least used climate policy instruments. In 2016, 18 countries and two Canadian provinces have implemented a carbon tax, with Chile set to do so in 2018 (Bloomberg New Energy Finance, 2016; Farid et al., 2016; World Bank, 2016). In comparison, 176 countries had policy targets for renewable energy and/or energy efficiency, and 110 national and subnational jurisdictions had a feed‐in tariff (REN21, 2017). Carbon tax proposals have been undone, sometimes at an advanced political stage, for example in Australia (in 2014), France (in 2000), Switzerland (in 2000 and 2015), and most recently in the United States in Washington State (in 2016). In other contexts, policymakers may have simply refrained from including carbon taxes in their agenda. The underutilization of carbon taxes is striking and potentially a concern.

This perspective paper explores practical ways through which carbon taxes can be made more politically attractive. It provides an extensive review of the growing empirical evidence on people's attitudes toward environmental taxes (cf. Appendix) and, from these findings, draws lessons on publicly acceptable forms of carbon taxation. The premise is that carbon taxes can be made more acceptable by designing them in a way that responds to voter concerns. Objections to carbon taxation are often not about the introduction of the tax itself, but about its design (Dresner, Dunne, Clinch, & Beuermann, 2006) and the way relevant information is shared. Sociopsychological factors—such as perceived coerciveness, equity, and justice—all affect the extent to which voters accept different climate policy instruments (Drews & van den Bergh, 2015). Factoring them into the design from the outset could make carbon tax legislation easier to pass.

It should be noted that popular opposition to a carbon tax is not the only reason for the limited diffusion of this instrument. Opposition by vested interests has proved to be very effective in limiting public intervention in a wide range of environmental issues (Oates & Portney, 2003), and their lobbying efforts can influence voters' views, preventing the passage, or even revoking the implementation of a carbon tax. In this respect, Australia is a prominent example of how regulatory capture can postpone the implementation of carbon pricing schemes, and once a scheme is eventually implemented, exert, successfully, its power to revert to a situation of unambitious climate policy (see Crowley, 2013; Spash & Lo, 2012). Other studies, for instance by Hammar, Löfgren, and Sterner (2004), Van Asselt and Brewer (2010), Dechezleprêtre and Sato (2017), and Neuhoff et al. (2015), provide insights into how vested interests and other political economy aspects have affected the design of carbon pricing in recent times.

In contrast, this paper focuses on *public* acceptability: addressing this lies in the hands of governments, and of public interest groups supporting effective climate policy. We present a set of stylized facts and discuss strategies that can enable a transparent and open debate on the implications of implementing a carbon tax. These strategies may contribute to addressing the potential concerns of voters, which may be inflated by the communication strategies of energy‐intensive industries. Advocacy groups, and the scientific community, may also contribute to ensuring an informed debate.

The remainder of the paper is organized as follows. Section 2 presents the general attitudes toward carbon taxes. Section 3 focuses on the level of stringency. Section 4 focuses on the use of revenues. Section 5 provides suggestions to policymakers. Section 6 concludes.

## 2 GENERAL ATTITUDES TOWARD CARBON TAXES

The reluctance of policymakers to adopt carbon taxes, and their preference for other policy instruments, reflect at least in part the attitudes of their country's voters (Hsu, 2011). Different quantitative and qualitative studies show people's preference for low‐carbon subsidies over taxes (Cherry, Kallbekken, & Kroll, 2012; de Groot & Schuitema's, 2012; Kallbekken & Aasen, 2010; Steg, Dreijerink, & Abrahamse, 2006). A survey on American citizens by Leiserowitz, Maibach, Roser‐Renouf, Feinberg, and Rosenthal (2013) found that while 71% of the American public support tax rebates for energy‐efficient vehicles or solar panels, only 43% would support a carbon tax, even if assumed to cost the average American household the relatively low amount of US$180 per year. That is, voters tend to prefer subsidies and tax rebates to carbon taxes. However, the evidence is more equivocal on regulation, and the extent to which it is preferred to carbon taxes (see Cherry et al., 2012; Clinch & Dunne, 2006; Deroubaix & Lévèque, 2006; Steg et al., 2006).

The stylized facts that we discuss in this paper, and the suggestions that we derive from them, are based on general trends across different samples collected in various countries. There is, of course, much heterogeneity across individuals in how climate change is perceived, and in how preferences for public intervention are defined. Drews and van den Bergh (2015) provide an extensive survey of this heterogeneity, focusing principally on socioeconomic and psychological factors. A recent paper by Cherry, Kallbekken, and Kroll (2017) suggests that deeper cultural aspects such as worldviews may also play a role in explaining aversion to policy interventions in general, as well as to some specific instruments. For instance, their study finds that people who are more hierarchical and/or individualistic are more averse to policy interventions than those who are more egalitarian and/or communitarian. Instruments that are perceived as coercive are more “offensive” to individualists, while instruments that include income redistribution are more attractive to egalitarian types.

Recognizing that there are variations in attitudes and perceptions across individuals, we identify five general reasons for aversion to carbon taxes that have been recurrently emphasized in the literature.

*Concern 1: The personal costs are perceived to be too high.* There is a perception among voters that the personal costs of a tax would be too high. A Swedish survey by Jagers and Hammar (2009) found that people associate carbon taxes with higher personal costs, more than they do with alternative policy instruments. A discrete choice experiment by Alberini, Scasny, and Bigano (2016) showed that Italians had a preference, among climate policy instruments, for subsidies over carbon taxes. Participants in a lab experiment by Heres, Kallbekken, and Galarraga (2015) similarly expected higher payoffs from subsidies than from taxes, especially when there was uncertainty on how tax revenues would be “rebated.” Ex ante, individuals tend to overestimate the cost of an environmental tax, and underestimate its benefits (Carattini et al., 2018; Odeck & Bråthen, 2002; Schuitema, Steg, & Forward, 2010). They are also prone to ignore the indirect costs of subsidies, which will most likely be financed through either higher income taxes or higher electricity bills (Jagers & Hammar, 2009; Kallbekken & Aasen, 2010). The literature in social psychology also suggests that individuals prefer subsidies because they are perceived as less coercive than taxes. Taxes are “pushed” onto polluters, imposing a mandatory cost, while subsidies are seen as “pull” measures, which supposedly reward climate‐friendly behavior (de Groot & Schuitema, 2012; Rosentrater et al., 2012; Steg et al., 2006).

*Concern 2: Carbon taxes can be regressive.* Many voters object to the regressive nature of carbon taxes. They perceive, rightly, that without counterbalancing measures carbon taxes may have a disproportionate negative impact on low‐income households. These counterbalancing measures can, however, offset the adverse distributional effects of carbon taxes, and even make them progressive. Furthermore, it is important to keep in mind that alternative climate policy instruments such as subsidies for renewable energy can also have similar regressive effects and may not generate revenues to counter them (Baranzini et al., 2017).

*Concern 3: Carbon taxes could damage the wider economy.* People are concerned about the wider economic impact of a carbon tax. This has been illustrated in Switzerland, where, in two different instances more than 10 years apart, concern about the potential competitiveness and employment effects of energy taxes contributed to their rejection in public ballots, even in the context of very limited unemployment (Carattini, Baranzini, Thalmann, Varone, & Vöhringer, 2017; Thalmann, 2004). While these concerns are partly justified, voters may tend to overestimate competitiveness and job effects. In the specific case of Thalmann (2004), for instance, virtually all respondents expressed concern for unemployment, despite there were no unemployed individuals in the sample and the population‐wide unemployment rate was, at the time in Switzerland, below 2%. Given that the proposals in the ballot were not especially disruptive, and given that most people in the sample were unlikely to be seriously exposed to unemployment risks, we consider this instance as a case of overreaction. Fears of competitiveness effects and job losses may also result from specific information campaigns led by energy‐intensive companies, as in the case of Australia (cf. Spash & Lo, 2012).

*Concern 4: Carbon taxes are believed not to discourage high‐carbon behavior.* Individuals do not see carbon taxes as an effective way to discourage high‐carbon behavior (Klok, Larsen, Dahl, & Hansen, 2006; Steg et al., 2006). They consider low‐carbon subsidies to be a more powerful way to reduce greenhouse gas emissions, especially if the cost of switching from consuming high‐carbon goods to low‐carbon goods is considered high. Many voters believe that the price elasticity of demand for carbon‐intensive goods is close to zero. The expectation that carbon taxes do not work is one of the main reasons for their rejection by people in surveys and real ballots (Baranzini & Carattini, 2017; Carattini et al., 2017; Hsu, Walters, & Purgas, 2008; Kallbekken & Aasen, 2010; Kallbekken & Sælen, 2011).

*Concern 5: Governments may want to tax carbon to increase their revenues.* The final reason for opposition is that individuals are often suspicious of government motives. They assume—as a direct consequence of concern 4 above—that the purpose of introducing a carbon tax is not to reduce greenhouse gases but to increase government revenues (Klok et al., 2006). At its core, this is an issue of trust. Trust issues sometimes concern the specific environmental tax proposal under consideration, but they may also be broader, related to people's general view of tax policy or even to trust in the government itself (Baranzini & Carattini, 2017; Beuermann & Santarius, 2006; Dietz, Dan, & Shwom, 2007; Hammar & Jagers, 2006).

Some of these perceptions are incorrect. There is evidence that carbon pricing does in fact reduce emissions (J. Andersson, 2015; Baranzini & Carattini, 2014; Martin, de Preux, & Wagner, 2014) and has so far had a minimal impact on the wider economy, in terms of adversely affecting the competitiveness of domestic industry, at least in the presence of adjustments and specific measures tailored to support the most exposed firms (Dechezleprêtre & Sato, 2017). On the other hand, voters are right to suspect that governments would probably welcome the extra revenues. Indeed, its benign fiscal implications are often highlighted as one of the merits of a carbon tax (Bowen & Fankhauser, 2017). It is also the case that carbon taxes are often regressive; without counter measures they may affect poor households disproportionately (Gough, Abdallah, Johnson, Ryan Collins, & Smith, 2012; Metcalf, 2009; Speck, 1999; Sterner, 2011). From a public acceptability perspective, the accuracy of public perceptions is less important than the fact that they are widely held and can hinder the adoption of otherwise desirable policies. Policymakers, however, are encouraged to strive for the design that minimizes the cost for society, while ensuring it receives sufficient public support. Understanding public perceptions is a first step in this direction.

## 3 ATTITUDES TOWARD TAX RATES

When designing a carbon tax, a key decision is the level at which the tax should be set and how it may evolve over time. Is it better to start with a high tax rate that remains fairly constant over time, or to increase tax levels gradually? Climate change economists usually recommend a carbon tax that increases over time, since this aligns with the prospect of an increasingly tighter carbon constraint. The required tax level is determined by the environmental objective and more specifically by the marginal costs of meeting a given emissions target (Bowen & Fankhauser, 2017). A rising schedule may raise concerns about a possible response from fossil fuel producers, who could accelerate fossil fuel extraction in anticipation of higher taxes (the so‐called Green Paradox). However, following Hotelling's rule, a Green Paradox would only occur if the tax rate was to rise too fast, and in particular if its growth rate was constantly above the interest rate (van der Ploeg & Withagen, 2015).

### 3.1 The impact of tax level on attitudes

It is a standard tenet of public choice theory that people do not like high taxes. It is not immediately obvious, however, that the same sentiment should extend to environmental taxes such as those on carbon. Environmental taxes are Pigovian taxes (after Pigou, 1920), put in place to correct a market failure. Voters may accept them on the grounds that they address a problem people care about. Yet empirical studies consistently find that the standard objection to high taxes also holds for carbon taxes (Brännlund & Persson, 2012; Carattini et al., 2017; Gevrek & Uyduranoglu, 2015; Sælen & Kallbekken, 2011; Thalmann, 2004). People's attitudes to carbon taxes appear to be influenced more by the direct personal cost of the measure than by an appreciation of the environmental objective (Kallbekken, Kroll, & Cherry, 2011).

Consequently, the public acceptability of an environmental tax depends heavily on its policy stringency, since the proposed tax rate determines the direct costs to consumers. Aversion to higher tax rates is even found when revenues are redistributed to the population. That is, voters tend to dislike sudden changes to taxation even if, on paper, these may not make them worse off.

The effect of tax levels on acceptability can be measured relatively precisely with choice experiments. For instance, Sælen and Kallbekken (2011) assessed the acceptability of fuel taxes in Norway, analyzing the responses of 1,147 survey participants. Brännlund and Persson (2012) studied the acceptability of carbon taxes with a survey of 2,400 Swedish citizens. Gevrek and Uyduranoglu (2015) surveyed 1,252 individuals from 16 Turkish cities about their attitude to a carbon tax. All three studies found that the acceptability of a tax proposal decreases with the personal cost it would impose on survey respondents.

**Figure 1:**
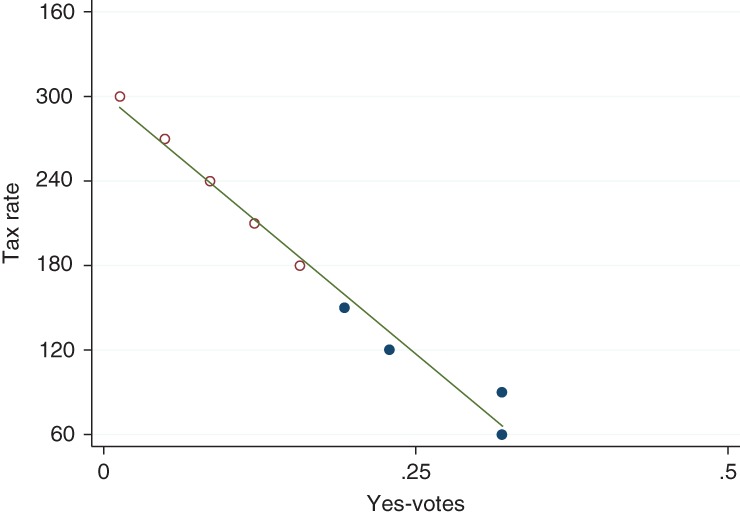
Predicted percentage of votes in favor of a carbon tax, by carbon tax rates. *Note.* Filled circles indicate observation in the sample; empty circles indicate observations obtained through extrapolation. In this scenario, the choice experiment assumed that revenues would be used to reduce the value‐added tax, as in the referendum case. (Reprinted with permission from Carattini et al. (2017). © The Author(s) 2017 Open Access. This article is distributed under the terms of the Creative Commons Attribution 4.0 International License (http://creativecommons.org/licenses/by/4.0/))

Two Swiss studies have used voter surveys to analyze the drivers of public opposition in public ballots. Thalmann (2004) analyzed the responses of a representative sampling of 990 Swiss residents after a referendum in the year 2000 on three different energy tax proposals, all of which were rejected. While the magnitude of the tax rate was not a decisive factor for most voters, Thalmann showed that it was important to a fraction of voters with a particular concern about the cost of the tax. Carattini et al. (2017) analyze another tax proposal, put forward in 2015, which was rejected by 92% of voters. The proposal entailed a tax swap in which a new energy tax on nonrenewable energy would have generated the same revenues as value‐added tax, which would have disappeared completely. The complete replacement of the value‐added tax, and the constraint to keep revenues constant over time, would have implied a high, and growing, tax rate. This concern, and its implications for distributional and competitiveness effects, led, among other factors examined in the paper, to the massive rejection in the ballot. To analyze how alternative tax designs could have performed in a ballot, Carattini et al. (2017) administered a second survey, a choice experiment with a representative sample of 1,200 Swiss voters. The researchers found that the acceptability of the tax almost linearly decreased as the tax rates increased (see Figure 1). They also found that people with low levels of climate change concern showed a higher sensitivity to tax rates, while people with stronger climate change concern paid less attention to price levels.

### 3.2 The impact of time and experience on attitudes

There is evidence that public opposition to high Pigovian tax rates may not be persistent. Instead, voter aversion may abate once a policy is implemented, as people become more familiar with the measure and are better able to gauge its costs and benefits. This is important as it suggests that, under favorable circumstances, Pigovian tax rates can ultimately be raised to the environmentally appropriate level.

The best evidence on the ability of voters to update their beliefs comes from studies of congestion charges and taxes on waste. Hensher and Li (2013) reviewed the difference in the ex ante and ex post acceptability of congestion charges in London, several cities in Norway, and Stockholm. In Stockholm, people voted in a referendum after trialing a congestion charge. The review suggests that a large proportion of survey participants in these cities would have rejected the congestion charge prior to its introduction. However, many of them changed their mind once they saw the effectiveness of the tax in reducing road usage and felt the benefit of reduced congestion (see also Börjesson, Eliasson, Hugosson, & Brundell‐Freij, 2012; Eliasson & Jonsson, 2011; Odeck & Bråthen, 2002; Schuitema et al., 2010; Winslott‐Hiselius, Brundell‐Freij, Vagland, & Byström, 2009). People also learned that the perceived costs of the charge were smaller than expected, and not greater than the personal and social benefits (Schuitema et al., 2010). There is also evidence from the Swedish city of Gothenburg, which also organized a trial for its congestion charge, to suggest that acceptability increased with experience. The policy was still rejected in the subsequent nonbinding referendum, but survey data showed that acceptability would have been 5% lower if voters had expressed their opinion before the trial (Hansla, Hysing, Nilsson, & Martinsson, 2017). Another survey shows that, once the policy was in place, both commuters and noncommuters were less skeptical about it (D. Andersson & Nässén, 2016).

Carattini et al. (2018) exploited a regulatory change in a region of Switzerland, which enabled them to analyze people's perceptions and acceptance of pricing domestic refuse by the bag before and after the scheme's implementation, and to compare them with a control group composed of people living in municipalities that had already implemented the tax. Their study suggests that residents were willing to pay 70% more for the price of a refuse bag once the policy was implemented than they had been before. They perceived the tax to be much more effective and fair once it had been applied.

In a more generic study, Cherry, Kallbekken, and Kroll (2014) designed a lab experiment in which participants in some treatments were given the chance to experience a Pigovian tax during a trial period, before voting on how to address the negative impacts of their own action on others. Trial runs were shown to help participants overcome, at least in part, their aversion to Pigovian taxes.

An important caveat needs to be introduced at this stage. It is difficult to extrapolate the findings from one policy area (transport, waste) and apply them to another (carbon taxation). The issues differ markedly in the ease with which benefits can be made visible to voters and in the salience of policy effects on behavior, with the positive impacts of a congestion or waste charge being much more immediately obvious than those resulting from a carbon tax. That said, there is some encouraging evidence on carbon taxation from the Canadian province of British Columbia. British Columbia's carbon tax was set at a tax rate of C$10 per ton of CO_2_ equivalent (tCO_2_e) when implemented in 2008, and then increased by C$5 per tCO_2_e each year up to C$30 per tCO_2_e in 2012. Murray and Rivers (2015) show with poll data that despite this threefold increase in the tax rate in just 7 years, public support for the tax grew from relatively low support to moderate support, and surpassed 50% in 2011, 3 years after the tax was implemented.

## 4 ATTITUDES TOWARD THE USE OF TAX REVENUES

Another defining feature of a carbon tax is how its revenues are proposed to be spent. Fiscal experts would recommend that tax proceeds should be treated as general government revenue. This method enables governments to optimize the tax system as a whole and incorporate climate change into overall tax and spending decisions, alongside other public policy concerns (Bowen, 2015).

However, empirical studies show that, against the wishes of experts, public acceptance for a carbon tax is higher if the use of proceeds is clearly specified. The literature has explored three revenue recycling strategies in particular: the earmarking of revenues to support emission reduction projects, the redistribution of revenues to achieve a fairer (less fiscally regressive) outcome, and the reduction of other taxes to achieve a revenue‐neutral outcome.

In investigating which strategies are most popular, an early set of studies used focus groups to gather people's opinion. These focus group studies, which were conducted in Denmark (Klok et al., 2006), France (Deroubaix & Lévèque, 2006), Germany (Beuermann & Santarius, 2006), Ireland (Clinch & Dunne, 2006), and the United Kingdom (Dresner, Jackson, & Gilbert, 2006), all showed that earmarking energy tax revenue to support further emissions reductions was the most preferred option for their participants, followed by social cushioning measures to help vulnerable groups, such as low‐income households and those living in remote areas. Revenue‐neutral forms of redistribution implying a reduction in existing taxes were the least preferred option for recycling tax revenues.

Three key options for revenue recycling, and the reasons for their popularity, are explored in what follows. While considering these options, policymakers are encouraged to also evaluate their cost, as all of them diverge from the “first‐best” tax designs advocated by economists.

### 4.1 Earmarking proceeds

The attractiveness of earmarking carbon tax revenues has been established in a range of contexts (cf. Baranzini & Carattini, 2017; Beuermann & Santarius, 2006; Bristow, Wardman, Zannia, & Chintakayalab, 2010; Carattini et al., 2017; Clinch & Dunne, 2006; Deroubaix & Lévèque, 2006; Dresner, Jackson, & Gilbert, 2006; Gevrek & Uyduranoglu, 2015; Kallbekken & Aasen, 2010; Kallbekken & Sælen, 2011; Klok et al., 2006; Thalmann, 2004). The same preference also holds in other areas of environmental policy. For example, Beuermann and Santarius (2006), Garling and Schuitema (2007), and Odeck and Bråthen (2002), find that the acceptability of congestion charges and fuel taxes increases if revenues are used to improve public transport.

The interest in earmarking reflects two voter concerns. The first is a lack of trust in government: voters do not trust politicians to make good use of revenues, if not specifically earmarked or redistributed back to the population (Beuermann & Santarius, 2006; Deroubaix & Lévèque, 2006; Hammar & Jagers, 2006). The second concern is doubt about the effectiveness of carbon taxes (as explained in Section 2 above). Using tax revenues for additional emissions reduction reassures voters that the tax will be effective and the environmental objective will be met (Baranzini & Carattini, 2017; Kallbekken et al., 2011; Sælen & Kallbekken, 2011).

Earmarking signals to the public that efforts are being made to make low‐carbon options both technologically and commercially more viable and so will reduce the personal cost of changing behavior (Kallbekken & Aasen, 2010). Earmarking is also seen as a potential solution to a perceived underinvestment in low‐carbon research and development. For example in a study for Vancouver, Hsu et al. (2008) found that individuals were willing to increase fuel tax rates if the revenue was earmarked for environmental technologies. Similarly, Sælen and Kallbekken (2011) found in a Norwegian study that earmarking tax revenues for environmental purposes (such as supporting public transport, construction of bicycle and footpaths, noise screening, or development of clean technologies) garnered majority support to increase fuel taxes by up to 15%.

It should, however, be noted that earmarking revenues for environmental purposes may not be a universal solution. A Swedish survey conducted by Jagers and Hammar (2009) showed that respondents were unwilling to increase carbon tax rates, as they felt the carbon taxes they paid on transport fuels were high enough already. Respondents preferred alternative policies such as decreasing taxes on clean energy sources, expanding public transport, and increasing information campaigns about vehicles' contribution to climate change. In the same vein, residents in Edinburgh considered that the public transport system was already well funded and functioning when a congestion charge with revenues to be earmarked for public transport was proposed to them (Gaunt, Rye, & Allen, 2007).

Additional evidence suggests that preferences for revenue recycling may be context dependent. In their discrete choice experiment on Swiss energy taxes, Carattini et al. (2017) found that providing information about the environmental effectiveness of different carbon tax designs reduces the preference for environmental earmarking. The authors used modeled results of the impacts on the wider Swiss economy of different carbon tax designs to inform respondents about the greenhouse gas abatement achieved under different combinations of tax rates and revenue recycling rules. Under all scenarios, a carbon tax produced a reduction in greenhouse gas emissions. Providing this information reduced voters' doubt about the effectiveness of carbon taxes, and in turn reduced the demand for environmental earmarking.

### 4.2 Compensating low‐income households

A second important approach to revenue recycling is the use of tax proceeds to compensate potential losers. Several strategies have been put forward in the literature to address potential adverse distributional effects of a carbon tax, including in the influential perspectives of Speck (1999), Baranzini, Goldemberg, and Speck (2000), and Metcalf (2009). In what follows, we cover what we consider the main options, based on the literature and on actual policymaking.

We begin with lump‐sum transfers. Compensation via lump‐sum transfers is progressive because fixed amounts of compensation account for a greater proportion of income in low‐income households. Since low‐income households tend to spend less, in absolute terms, on energy consumption than their high‐income counterparts, carbon taxes with lump‐sum transfers tend overall to be progressive: that is, low‐income households are likely to receive compensation from the government amounting to more than the cost increase that they suffer. If all tax revenues are redistributed back to the population, a carbon tax with lump‐sum transfers represents a revenue‐neutral reform.

Another option is social cushioning. Social cushioning is purposely designed to be progressive by providing lower‐income households with a higher amount of the tax revenue, for instance through an especially generous income tax rebate or through targeted lump sum transfers. Social cushioning measures have been used for instance in Alberta and British Columbia, Canada, (Carl & Fedor, 2016). Further ways to design carbon taxes to make the outcomes progressive, and hence more socially acceptable, are shown in Table 1.

**Table 1:** Ways a carbon tax can be designed to be progressive

Design option	Mechanisms
Differentiated tax rates	Threshold taxes: Consumption of carbon below a certain level is exempt from a carbon tax, which, in practice, is equivalent to redistributing part of the revenues through lump‐sum transfers (e.g., if the threshold is 4 tons of CO_2_ and the price is $40/tCO_2_, $160 would be redistributed to make the first 4 tons “free”)
Revenue recycling	Lump‐sum transfers, distributed across households in equal shares (per capita) Lump‐sum transfers, distributed across eligible households, with eligibility depending on, for example, household income (e.g., Alberta, Canada, provides lump‐sum transfers only to households below a given income threshold) Lump‐sum transfers whose amount is defined based on equivalence scales (e.g., Alberta gives less weight to children or the second adult when redistributing revenues across eligible households) Subsidies/grants for low‐carbon technologies, with eligibility restricted to low‐income households Subsidies for low‐carbon options that low‐income households are more likely to use (e.g., public transport)
Other social cushioning measures	Subsidies to compensate low‐income households (paid through general budget), not necessarily tied to low‐carbon consumption (e.g., food stamps)

Everything else equal, the literature finds a positive relationship between progressivity and acceptability. People seem to value tax schemes that are perceived to be fair and that create a lighter burden for low‐income households. Distributional issues are a constant feature of qualitative studies, as they are virtually always brought up by interviewees (e.g., Beuermann & Santarius, 2006; Clinch & Dunne, 2006; Deroubaix & Lévèque, 2006; Dresner, Jackson, & Gilbert, 2006; Kallbekken & Aasen, 2010). The importance of distributional effects is also confirmed in quantitative surveys (e.g., Baranzini & Carattini, 2017; Kallbekken & Sælen, 2011). Choice experiments are especially well designed to test the acceptability of different features of a carbon tax, including their effect on low‐income households. Bristow et al. (2010) started to analyze people's preferences for progressive cost distributions by testing the acceptability of a tax threshold (cf. Table 1), which received relatively strong support from survey respondents. Designs that are explicitly presented to the respondent as progressive, as in Brännlund and Persson (2012) and Gevrek and Uyduranoglu (2015), also perform better than neutral or regressive designs, everything else being equal.

However, the literature suggests that when there is a clear trade‐off in the use of revenues between environmental earmarking and socially progressive redistribution forms, people tend to prefer to use revenues for environmental earmarking (Baranzini & Carattini, 2017; Sælen & Kallbekken, 2011). The misperception that a carbon tax by itself will not lead to a reduction in emissions seems to be the dominant concern; before giving their support, people want to be sure that the carbon tax leads to lower emissions. Nevertheless, concerns over the distribution of impacts are likely to persist, and at the margin may make the difference between a successful policy and one that is rejected. However, policymakers can reduce the demand for environmental earmarking by providing information on the effectiveness of the planned carbon tax. In the study by Carattini et al. (2017), the most favored options for using revenue were redistribution through lump‐sum transfers, and social cushioning. In the setting of this study, information was provided to respondents also on the distributional effects of each design. Social cushioning represented, by design, the most progressive redistribution form. Thanks to the information that they received, people also realized the favorable distributional properties of lump‐sum transfers, which may not otherwise be evident to the general public.

### 4.3 Cutting other taxes

The third main strategy for the use of tax proceeds is to cut other taxes and secure in this way full or partial revenue neutrality. Empirical studies show that cutting other taxes is the least popular redistribution strategy among the public (Beuermann & Santarius, 2006; Dresner, Jackson, & Gilbert, 2006; Klok et al., 2006; Thalmann, 2004). This is in contrast to many economists, for whom using tax revenues to reduce distortionary taxes is the ideal solution. By using carbon tax revenues levied on “bads,” such as greenhouse gas emissions, to reduce distortionary taxes on labor, profits, or consumption, which discourage desirable activities, one can hope to achieve higher economic output on top of emissions abatement, and so obtain a “double dividend” (cf. Goulder, 1995).

Qualitative studies offer possible explanations for people's resistance to this third strategy. One reason for public opposition is that voters do not necessarily buy into the logic behind the double dividend. Focus groups with voters in Denmark (Klok et al., 2006), Germany (Beuermann & Santarius, 2006), Norway (Kallbekken & Aasen, 2010), and the United Kingdom (Dresner, Jackson, & Gilbert, 2006) show that people do not make the link between a policy that is aimed at reducing greenhouse gas emissions and the desire to reduce taxes in a different area. They perceive these to be separate problems requiring separate solutions. Sælen and Kallbekken (2011) describe this cognitive dissonance as an “issue‐linkage” problem.

Another reason for public opposition is a lack of trust in politicians and fiscal authorities (Hammar & Jagers, 2006). Even if people understand how a revenue‐neutral carbon tax would work, they may not believe that the government will actually implement these tax shifts (Klok et al., 2006). This puts the onus on the tax authorities to introduce commitment devices that reassure the public that the promised use of revenues would be maintained. Once the policy is implemented, governments could use information devices to increase the visibility of the tax shift. Compensation can be made visible by displaying the amount of income that is rebated on payslips, tax slips, or in contributions to social insurance (Clinch, Dunne, & Dresner, 2006; Dresner, Dunne, et al., 2006; Hsu et al., 2008).

## 5 POLICY IMPLICATIONS AND SUGGESTIONS

The growing empirical understanding of public attitudes toward environmental taxation can enable policymakers to design carbon taxes in a way that is more acceptable to voters. Below we offer some concrete design options that appear particularly promising to increase public support. While fairly prescriptive, these are high‐level suggestions that policymakers will have to adjust to their own political economy context. The options all diverge from the “first‐best” tax designs advocated by economists and therefore require a trade‐off between the theoretically desirable and the practically feasible. That is, while these suggestions may make implementing a carbon tax easier, they all come with an efficiency cost. Our objective is not to distract policymakers from their pursuit of cost‐effectiveness. Our purpose is to increase the probability that a carbon tax is passed into legislation, in a world in which still too often carbon taxes are rejected, or do not emerge, because of lacking public support. Hence, in our perspective, an imperfect carbon tax may still be better than no carbon tax at all. That said, we encourage policymakers to strive, everything else equal, for the design that minimizes the cost for society. Policymakers should not accept, passively, the existence of information asymmetries and biased perceptions. Policymakers should take a proactive stance and address these asymmetries. We provide specific suggestions in this respect.

Some of the options that we consider in the following sections may be implemented in conjunction; others are mutually exclusive. Regardless of which are used, the proposed carbon tax will require extensive information sharing and careful communication, both before and after implementation, to build continued trust and credibility. We review the suggestions in turn.

### 5.1 Phasing in carbon taxes over time

By phasing in carbon taxes gradually, policymakers can take advantage of the fact that aversion tends to abate once people have experienced a policy. A slow ramp‐up, or even a trial period, provides individuals with the opportunity to gauge the costs and benefits of the tax. Taxes can then be raised progressively until they reach the level required to meet the environmental objective. Note that this may imply renouncing to allowing the carbon tax rate to fluctuate depending on the business cycle, although this type of flexibility might be welfare improving (cf. Doda, 2016).

The risk with this strategy is that carbon taxes may be frozen at a level that is not sufficient to achieve their intended objectives. For example, the United Kingdom carbon price support, a tax on carbon emissions to ensure a minimum carbon price for UK facilities participating in the EU emissions trading system, was introduced in 2013 at £16 per ton of CO_2_ and was expected to steadily increase over time, up to £30 by 2020. Since 2015, the tax has, however, remained constant at £18.08, despite the original commitment to increase it further (Ares & Delebarre, 2016).

There are two potential, and complementary, solutions to overcome this risk. The first solution relies on societal learning. The second solution uses commitment devices.

Societal learning about the exact costs and benefits of the tax can overcome potential resistance since public acceptability tends to increase the more experience people have with carbon taxes. It is important that governments provide detailed information on the achieved reductions in greenhouse gases, but also that they highlight local cobenefits such as reduced congestion and improved air quality.

Commitment devices can ensure that initially low‐carbon tax rates will escalate toward the rate required to meet greenhouse gas reduction objectives. Commitment devices can provide clarity about the long‐term direction of travel, and reducing the risk that tax rates become subject to political cycles. The most common device is declaring explicit tax schedules to raise carbon tax rates. In Canada, the province of British Columbia introduced its carbon tax rate at C$10/ton CO_2_ in 2008, and successfully increased it by C$5/ton CO_2_ equivalent (tCO_2_e) each year to eventually reach C$30/ tCO_2_e in 2012 (Murray & Rivers, 2015). France has a more ambitious long‐term program: it introduced the carbon tax rate in 2014 at €7/tCO_2_e, committing to increase the rate by €8.50/tCO_2_ per year to reach €56/tCO_2_ in 2020, with further plans to increase it to €100/tCO_2_ in 2030 (World Bank, 2016). Switzerland sets emissions objectives in its CO_2_ Act (Nachmany et al., 2015). If predefined intermediate objectives for the emission reduction pathway are not met, the Swiss carbon tax rate, which covers only thermal fuels, can be increased by the government without consulting the legislator (Baranzini et al., 2017). For instance, the Swiss government was legally entitled to increase the tax rate from CHF60 to CHF84/tCO_2_e in 2016, as Switzerland underperformed on its emission reductions (1 Swiss Franc/CHF is approximately equal to 1 US dollar.)

### 5.2 Earmarking tax revenues for additional climate change mitigation

Voters have a preference for earmarking tax revenues and using the proceeds for additional greenhouse gas emissions reductions. They are particularly keen on support for low‐carbon research and development, along with subsidies to promote deployment.

Earmarking—or, in fiscal jargon, hypothecation—also responds to the widely‐held perception that on their own, carbon taxes are not effective. People tend to overestimate the costs of switching from high‐carbon to low‐carbon options. They believe additional government support to help them reduce emissions is necessary.

The demand for environmental earmarking may decrease over time as people observe the impact of the tax and update their beliefs. Governments can again support this process by providing effective information about emissions trends, the distributional effects of the tax, and any ancillary benefits. Revenues may then be freed up gradually to address other sources of voter aversion, or to obtain economic gains.

Tapering the degree of earmarking can also allay a government's concerns about fiscal management. A telling example is the French carbon tax, which was introduced in 2014: in the first year, 100% of revenues were dedicated to green transition plans, but this level of earmarking declined over time, to 44% in 2015 and 38% in 2016, with the remaining proportion of tax revenue going to general funds (Carl & Fedor, 2016; World Bank, 2016). The earmarking of tax revenues is controversial among fiscal experts because it complicates fiscal management. Earmarking commits the government to spending specific amounts of money on reducing emissions, even if there may be a poor match between actual spending needs and the revenues raised (Goulder & Parry, 2008).

### 5.3 Redistributing taxes to improve fairness

Carbon taxes can be made more acceptable if tax revenues are used to address important societal concerns. In surveys, individuals generally prefer to use revenues for environmental purposes, but do not dislike using them to ease the impact on low‐income households, which is a source of concern for most of them. The scope for redistributing tax revenues could increase over time, as higher tax rates are phased in (per option 1) and as the demand for earmarking decreases (per option 2). While the objective of a carbon tax is to address the climate externality, and not to address the issue of raising inequalities, there may still be the expectation that carbon taxes are designed in a way that at least does not lead to a more unequal distribution.

Carbon taxes can be designed to be both revenue neutral and progressive through lump‐sum transfers and social cushioning measures to reduce costs for low‐income households. Some voters may, however, be suspicious about a government's long‐term commitment to redistribution. To allay those fears, governments can use commitment devices, such as explicit plans on how revenues are to be redistributed. For instance, the Ministry of Finance in British Columbia is required by law to make explicit its plans every year, which are then approved by the Legislative Assembly. Annual reports can make redistribution transparent by providing regular updates on how revenues are used. Redistribution can also be made directly visible to the general public, for instance, by issuing explicit rebate cheques to households and firms. In all cases, governments would maintain the option to lower other social security measures as a result of the implementation of a carbon tax. This option suggests that there is no perfect way to allay people's concerns. At the same time, we note that by lowering other social security measures, governments would likely need the support of the legislative bodies, which can be assumed, in the general case, to be attentive to the concerns of their constituencies.

### 5.4 Information sharing and communication

A final suggestion applies to all efforts to implement a carbon tax, regardless of the use of revenues, or level of stringency. It concerns information sharing. As soon as policymakers start considering the design of a carbon tax, they should provide detailed information (obtained through analysis and perhaps model simulations) to navigate the process of public consultations and to pre‐emptively address voter concerns. This disclosure would ideally occur before voters are called to a ballot, or before lawmakers consider a carbon tax bill in the parliament. Providing rigorous analytical information through different, trusted channels and devices may ensure that the public debate about the effects of a carbon tax is based on the best available evidence. Important analytical results governments (or a trusted and independent institution) may wish to share include:

- The greenhouse gas reductions likely to be achieved at the chosen rate, and those achieved if tax rates are increased over time.
- Any local cobenefits, such as reduced congestion, air pollution, and health costs, improved atmospheric visibility and quality of life.
- Expected variation in cost for the goods most likely to be affected by the tax.
- Expected impact on the income of the average household as well as of low‐income households.
- Expected impact on the economy, including potential job losses or gains, along with simple explanations on the dynamics leading to the emergence of a double dividend, if any.

This information would account for any measures undertaken to minimize negative impacts, including tax rebates, lump‐sum transfers, or other social cushioning measures. Both Carattini et al. (2017) and Klenert et al. (2017) argue that a good communication strategy that increases the visibility of the progressiveness of lump‐sum transfers can convince voters that these “dividends” can overcome fundamental issues of distributional fairness, political trust, and policy stability.

An interesting example of a communication strategy is offered by the Citizens' Climate Lobby (CCL), an interest group active in promoting the implementation of a carbon tax with lump‐sum redistribution of revenues in the United States as well as in other countries. One of the CCL's main activities is communicating the functioning of the carbon tax to the general public. Their strategy starts with the name that they give to their carbon tax proposal: “carbon fee and dividend.” Thanks to external studies, the CCL is able to communicate some general approximations of the effects of its proposal on a relatively large set of outcomes, including the amount of the “dividend” that households would receive thanks to the carbon tax. General equilibrium effects on jobs, and economic output, are also provided, with variation at the regional level. These findings come from two preliminary consulting studies. Nystrom and Luckow (2014) evaluate the effect of a carbon fee and dividend on different sectors and regions in the United States. Unmel (2016) examines household consumption expenditures along different dimensions (including income, race and location) and measures the potential effect of the carbon fee and dividend in a static environment.

Clear communication strategies can also help to counter some of the claims that opponents of the tax may put forward. The “industrial flight” argument, that is, that businesses could relocate if climate policy is too stringent, may resonate particularly strongly with the general public (Spash & Lo, 2012). If voters are able to correctly evaluate the competitiveness risks to which firms are exposed, they are more likely to support reasonable carbon tax rates and vote against unjustified exemptions. Deroubaix and Lévèque (2006) show, for instance, that focus groups in France thought it was unfair for industries to be exempted from the energy tax reform (ETR) in 2000, arguing that all polluters needed to pay for the tax. Participants in a similar focus group organized in Denmark argued for a system rewarding polluters based on their efforts to become greener (Klok et al., 2006), rather than on their exposure to foreign competition, which is the criterion that economists would use to define “optimal” exemptions (Martin et al., 2014).

Communication efforts need to continue once the policy is implemented. Perceptions of a carbon tax may improve over time. The evidence that we mentioned above comes, however, from British Columbia, where the local government is committed to providing regular information on the tax to the population. Without this type of device, perceptions—and awareness—of carbon taxes may remain unchanged. For example, a survey by Baranzini and Carattini (2017), administered in 2012, suggests that a surprisingly large proportion of the population may not be aware of the carbon tax on heating fuels that the Swiss government introduced in 2008. Even fewer people seem to be aware that the revenues from this tax are redistributed lump sum to households, through automatic reduction in mandatory health care bills, in which this information is reported in fine print. According to a survey by INFRAS (2015), only a quarter of the 1,012 respondents interviewed were aware of the mode of redistribution.

Because the effects of carbon taxes are often not visible, governments are encouraged to measure their effects regularly and inform their citizens about them transparently. The provision of annual reports that include plans on how revenues have been redistributed in the past and how they will be distributed in the future provides evidence of transparency, credibility, and commitment of a government to execute a carbon tax as originally intended. A world without carbon tax is by definition not observable, once the carbon tax is implemented. Communicating the effect of a carbon tax may therefore be difficult when greenhouse gas emissions increase from year to year, but would have increased even more without the tax. Communication strategies need to be adapted to the fact that the general public may have little familiarity with the empirical toolkit of policy evaluation. Similar adjustments may need to be undertaken also ex ante, if greenhouse gases are expected to increase. Communication strategies also need to be tailored to the context in which they are used. Who provides this information, and how it is framed, may matter for acceptability. Communication strategies may need to be adapted to the beliefs and worldviews of the targeted population (Cherry et al., 2017), and also take into account the potential implications of political polarization and bipartisan divides (Hart & Nisbet, 2012; Kahan et al., 2011).

## 6 CONCLUSIONS

Making carbon taxation more acceptable to the public is important because it is an effective way of incentivizing the reduction of greenhouse gas emissions. By putting a price on carbon, emitters are confronted with the environmental cost of their actions, and incentivized to manage their carbon output.

Economists prefer carbon pricing over subsidies because it is less prescriptive technologically, simpler to administer and does not draw on government budgets. They prefer carbon pricing over carbon regulation because it affords emitters the flexibility to find their own way of reducing emissions. There is an important place for both subsidies and regulation in climate change policy, and most countries rightly use a mix of instruments. Nevertheless, putting a price on carbon is an essential aspect of cost‐effective emissions reduction. Carbon taxes have a role to play even in jurisdictions that already have an emissions trading system, such as the European Union. They offer an effective way to reduce greenhouse gas emissions outside the trading scheme.

Voters are instinctively against new taxes, even if they are explicitly aimed at preventing environmental harm. They are doubtful about the effectiveness of a carbon tax, dislike its coercive nature, and are concerned about its impact on low‐income households. These perceptions are not necessarily all correct, but they matter, and they have made it difficult to pass carbon tax proposals in the past. However, there are practical options to overcome these constraints. If policies are well designed, they will be more acceptable to, and accepted by the public. There are also practical options to try to correct misperceptions, for instance on short‐term versus long‐term elasticities. If policies are well communicated, the necessary gap between what is optimal and what is politically feasible may close over time.

The suggestions in this paper are based on the current state of the literature. While drawing our suggestions, we hit in a few instances what we think is the frontier in this literature. The role of communication strategies seem, in particular, to have been largely neglected. Understanding under which conditions information asymmetries can be addressed is a crucial step to guide policymakers, and the general public, toward policies that are both acceptable and cost‐effective. Providing information in a randomized fashion, in different forms and from different sources, may represent a promising avenue for future research. A first step in this direction has been taken, for instance, by an emerging experimental literature on public support for domestic versus international greenhouse gas emissions abatements (Baranzini, Borzykowski, & Carattini, 2018; Buntaine & Prather, 2018; Diederich & Goeschl, 2018). Furthermore, the effect of information provision may vary depending on the method used. A better understanding of the relationship between the methodology used and the findings it produces, for instance based on a meta‐analytical approach, may inform the literature on the opportunities and limits of each methodological option. In this respect, we invite authors to disclose more fully their research design and the information that was provided to respondents, participants, or voters.

Our paper does not include specific sections on hybrid strategies mixing different revenue recycling options. While these are relevant in our context, they have been underexplored in the literature, plausibly to limit the cognitive load in surveys and experiments. While it is reasonable that scholars look for low‐hanging fruits first, the literature is now sufficiently mature to expect future papers to also tackle more complex policy designs and realistic situations.

## CONFLICT OF INTEREST

The authors have declared no conflicts of interest for this article.

## RELATED WIREs ARTICLE

https://doi.org/10.1002/wcc.462

## APPENDIX A 1 EMPIRICAL FINDINGS TESTING FACTORS AFFECTING THE ACCEPTABILITY OF CARBON TAXES

**Table:**

Study (listed in order of publication year)	Location, year and type of policy intervention tested	Methodological details	Empirical findings
Thalmann (2004)	**Where and when:** Switzerland, 2000. **Type of policy intervention:** Three ballot proposals for ETR that include green tax (with revenue recycling), energy conservation tax (revenue used to promote energy conservation and renewables), and solar initiative (tax revenue used for solar and energy efficiency use).	**Methodology:** Quantitative analysis of possible combinations of votes (yes, no, and abstention) for three proposals, and of turnout in the ballot. **Data collection: “**VOX” opinion surveys of 990 Swiss citizens.	**Explanations of acceptance of energy taxes:** Respondents were more accepting of energy taxes if they had leftist or green affinities, higher education levels, lived in cities, did not own cars, and were younger than 60 years old. This study demonstrates that the actual referendum had more “yes” votes because more educated people (who also were in favor of ETRs) participated in the vote, in comparison with the number of yes votes that were modeled based on answers of survey respondents. As the study included citizens who did not participate in the actual referendum, the study finds that “yes” votes would be fewer if the entire voting population participated in the referendum. The study also found subjective characteristics of political preferences and attitudes toward environmental protection were correlated. Those who valued environmental protection were more willing to accept government intervention, while those who valued wealth preferred markets to be self‐regulated. Concern for income inequality and unemployment did lower acceptance for ETRs. Only half the respondents were concerned about the former issue while almost all expressed concern for the latter, despite the very low unemployment rate at the time of the ballot. For most respondents, the tax rate was not a decisive factor in explaining rejection of energy taxes. However, the magnitude of the tax rate led to increased rejection in groups particularly concerned about the costs of energy taxes (e.g., multiple car owners). Although concern about income inequality did lower the acceptance rate of energy tax, it was not an important issue for the majority of respondents. **Use of carbon tax revenues:** Broad revenue recycling, including lowering labor taxes (in the case of this study, by reducing contributions to social security) did not make ETR more acceptable than earmarking tax revenues to support environmental efforts, with the former proposal obtaining 44.6% “yes” votes, and the latter 46.6%. Note, however, that a third proposal received much less support (31.9% of “yes” votes). This proposal was designed to earmark revenues for a narrow set of low‐carbon energy initiatives.
Beuermann and Santarius (2006)	**Where and when:** Germany, 2000–2001. **Type of policy intervention:** ETR introduced in 1999, which involved increasing fuel taxes and using fuel tax revenue to reduce pension contributions.	**Methodology:** Qualitative analysis of interviews and focus groups. **Data collection:** Interviews with policymakers and firms from five key industries, and five focus groups representing the general public.	**Use of energy tax revenues:** Trust in government played an important role in finding acceptable revenue‐neutral fuel taxes through the ETR, especially with regard to believing that governments would actually redistribute fuel tax revenue to lower pension contributions (instead of using tax revenue to increase the general budget), and believing government's results showing that revenue‐neutral fuel taxes had been effective in improving environmental and employment outcomes. People could see the increase in fuel costs but not the corresponding decrease in pension contributions in their pay or tax slips, creating a salience‐related problem. Even if people understood that the revenue‐neutral tax was meant to achieve the “double dividend” of decreasing emissions while increasing employment, they believed the effect was not real or that it was negligible. Respondents were more supportive of earmarking fuel tax revenues for making low‐carbon alternatives more affordable (e.g., public transport) as a more acceptable form of revenue recycling than keeping fuel taxes revenue neutral, as it reduces the perceived personal costs of the fuel tax.
Clinch and Dunne (2006)	**Where and when:** Ireland, before 2006. **Policy intervention:** Hypothetical fuel tax reform (keeping tax revenue neutral).	**Methodology:** Qualitative analysis of interviews and focus groups. **Data collection**: Interviews with businesses and policymakers, and eight focus groups (with eight members in each group split evenly between males and females) to represent the Irish public.	**Explanations of aversion to fuel taxes:** Businesses and some participants believed the fuel tax would increase net personal costs—Especially as individuals believed they were already overtaxed. Furthermore, elasticity of fuel consumption was perceived to be low, and therefore the tax was expected to increase fuel costs but not to change incentives to shift to low‐carbon options—although some focus group participants agreed they would change to low‐carbon options if the tax increased prices drastically. Loss of competitiveness and jobs and closure of factories were further concerns. Focus groups found regulation, higher standards, and enforcement, to be more viable mechanisms for achieving environmental protection. **Phasing in fuel taxes:** Considered important by businesses for allowing adjustment time for businesses and people. **Use of fuel tax revenues:** Businesses and focus group participants had a poor understanding of fiscal neutrality in the redistribution of tax revenues, which implied increasing fuel taxes (according to carbon content) and decreasing existing taxes. Most focus groups did not trust the government to redistribute tax revenues. Furthermore, a previous government had integrated many discrete taxes into a single income tax, and therefore participants did not want a new initiative under the word “tax” as they viewed this as rescinding the terms of the 1970s tax reform. The most favorable ways to recycle revenues were to earmark revenue for environmental purposes (e.g., for improved technology grants and support of improvements in energy efficiency, subsidized energy audits and renewable energy, and improving energy efficiency of buildings), and to reduce adverse distributional effects (e.g., with grants to improve energy efficiency for low‐income households and sectoral exemptions to industry most vulnerable to foreign competition). It also increased participants' trust that government would spend the tax revenue on the original environmental problem.
Deroubaix and Lévèque (2006)	**Where and when:** France, 1999–2000. **Type of policy intervention:** ETRs (with revenue recycled to lower labor tax). Implementation began in 1999 but was declared unconstitutional by the judicial court in 2000.	**Methodology:** Qualitative analysis of interviews and focus groups. **Data collection:** Interviews with policymakers and firms, and five focus groups representing the general public.	**Explanations of aversion to energy taxes:** Focus groups saw regulations as a more acceptable policy intervention as it prevented “free riding” as everyone had to adhere to the same standard. Taxes were seen as a way of allowing the wealthy to “pay to pollute.” Participants in the focus groups also preferred earmarking energy tax revenue for environmental purposes as doing so addresses the environmental problem, and increases confidence and transparency in how revenue is used. Other forms of revenue recycling, including keeping taxes revenue neutral, were considered with great suspicion.
Dresner, Jackson, and Gilbert (2006)	**Where and when:** United Kingdom, 2000–2001 (after policy announced but before implementation). **Type of policy intervention:** Revenue‐neutral measures of the Climate Change Levy (CCL—a carbon tax based on carbon content of fuels). The CCL was announced in March 1999, its final design defined in March 2000, and implemented in April 2001.	**Methodology:** Qualitative analysis of interviews and focus groups. **Data collection:** 10 interviews with policymakers, eight with businesses, and five focus groups.	**Aversion to environmental taxation generally:** People were not against environmental taxation outright, but more against the specific design of the CCL. **Aversion to revenue‐neutral fuel taxes:** Most focus group participants were skeptical that a redistribution of the revenues from the CCL would occur once the policy was in place. Nor did people understand the purpose of the tax shift, and this increased distrust in the government and generated suspicion that it would not redistribute the revenue. Focus groups did not see why recycling revenues from fuels should be used to “reward” reductions in labor taxes, or believed revenue‐neutral fuel taxes would not be effective in reducing emissions by changing the relative incentives between high‐ and low‐carbon goods. **Use of fuel tax revenues:** Focus group participants believed earmarking revenue for environmental purposes (particularly energy conservation) showed government commitment to reducing emissions. Such earmarking would be targeted at incentives for improving the environment.
Hammar and Jagers (2006)	**Where and when:** Sweden, 2002. **Type of policy intervention:** Existing carbon tax on transport fuels (with hypothetical scenario of increasing rates).	**Methodology:** Quantitative analysis of discrete choice experiment involving different attributes of carbon taxes on transport fuels, including increase in tax rates. **Data collection:** 1,270 responses to a mailed survey.	**Increasing tax rates:** Most of the samples were against increasing existing fuel tax rates, with only 21% of respondents in favor. However, findings show that increased confidence in the effectiveness of the carbon tax to reduce emissions increases support for raising the carbon tax. Therefore, information devices to demonstrate that carbon taxes have changed incentives to lower emissions are considered important to build support for increasing future taxes. **Explanations of aversion to increasing carbon taxes:** Trust in politicians is the most significant factor to support an increase in carbon tax rates, even within groups of similar people. Green party members who have high trust in politicians are more likely to support an increase in tax rate than those with low trust in politicians. Motorists who trust their politicians are not more likely to resist carbon tax increases than high‐trusting persons with no access to a car—suggesting that trust in politicians, rather than self‐interest, is the more important factor in understanding resistance to tax increases.
Klok et al. (2006)	**Where and when:** Denmark, no date provided. **Type of policy intervention:** Existing environmental tax reforms, implemented in Denmark in 1993 (involving taxing fuel, carbon, and water consumption to reduce labor taxes on firms).	**Methodology:** Qualitative analysis of interviews and focus groups. **Data collection:** Interviews with businesses from five key industries, and six focus groups representing the general public.	**Introducing and adjusting environmental taxes:** Focus groups showed less concern for global, and less visible, environmental problems. Focus group participants called for independent environmental authorities to provide information campaigns showing how environmental taxes have visible and objective environmental goals, prior to their introduction, and to provide continuous feedback showing progress on how these objectives are met once the tax is implemented. The tax can be adjusted according to how well objectives are met. **Use of carbon tax revenues:** Respondents believed environmental taxes were a backdoor way to increase the general budget rather than to change consumption incentives. Although Denmark has implemented revenue‐neutral environmental taxes since 1993, few believed the redistribution worked in practice as they had not seen reduction in labor taxes, nor were aware of any associated increase in employment. Those who had had concern for socially‐adverse effects preferred tax designs that provided compensatory measures, including using revenues for supporting low‐income and large families through subsidies or raised income tax thresholds, personal green allowances, or progressive tax rate systems. However, the most accepted use of revenues was earmarking for environmental purposes, including rewarding those firms/people who had put efforts into reducing their environmental impacts (e.g., through special tax reductions).
Steg et al. (2006)	**Where and when:** Groningen, Netherlands, 2003. **Type of policy intervention:**16 hypothetical pricing policies aimed at reducing household CO_2_ emissions.	**Methodology:** Quantitative analysis based on survey questionnaire testing psychological factors. The characteristics of these policies are emblematic of taxes (referred to as “push” policies in study), subsidies (referred to as “pull” policies), regulations (referred to as “curtailment”), and measures to promote energy efficiency. **Data collection:** 112 responses from mailed survey questionnaires.	**Explanations of aversion to/acceptance of carbon taxes:** People found subsidies more effective and acceptable than “coercive” measures such as taxes, even when taxes were perceived to increase the cost of high‐carbon behavior. Regulations that limit consumption were perceived less effective than measures that promote energy efficiency. **Use of carbon tax revenues:** Carbon taxes were seen to be acceptable and effective when tax revenues were earmarked to subsidize low‐carbon options, rather than to be recycled into general funds.
Dietz et al. (2007)	**Where and when:** Virginia and Michigan, USA, 2004. **Type of policy intervention:** Eight hypothetical policies proposed to reduce the burning of fossil fuels.	**Methodology:** Quantitative analysis based on survey questionnaire testing psychological factors predicting policy support for different hypothetical policy interventions. **Data collection:** Mailed survey responses from 316 Michigan and Virginia residents.	**Explanations of aversion to/acceptance of fuel taxes:** Trust in different actors (environmental institutions, industry, and government) played an important role in determining support for environmental action, with lowest trust in industry, and highest in environmental NGOs. **Preferred policy intervention:** Policies that increased the costs of fuel consumption, such as a gas tax, had the least acceptance. 75% of the sample supported shifting subsidies for fossil fuels to cleaner forms of energy.
Hammar and Jagers (2007)	**Where and when:** Sweden, no date provided. **Type of policy intervention:** Hypothetical increase of existing carbon tax on transport fuels.	**Methodology:** Quantitative analysis of survey questionnaire. **Data collection:** 932 responses from questionnaire mailed to a random sample of the Swedish population (with addresses drawn from national register).	**Explanations of aversion to/acceptance of increase in fuel taxes:** Those who did not have cars, or drove infrequently, were more inclined to support increasing the fuel tax, and believed that the polluters should pay for the pollution that they caused (that is, those who drive and pollute more should pay more). However, those who used cars frequently were more likely to favor distributing the costs of mitigation equally across the car‐driving population (that is, car drivers reduce pollution by the same amount, regardless of how frequently they drive). Therefore self‐interest motivates in part how people perceive which principle is the most fair in distributing the burden of climate policy.
Hsu et al. (2008)	**Where and when:** Vancouver, Canada, no date provided. **Type of policy intervention:** Existing gasoline tax with hypothetical suggestion to increase tax by C$0.5 per liter.	**Methodology:** Quantitative analysis of discrete choice experiment on increasing gasoline tax by C$0.5, and preferences for revenue use; expression of tax rebates in monetary or relative terms. **Data collection:** Face‐to‐face surveys in public places in Vancouver, with 797 responses.	**Explanations of aversion to/acceptance of fuel taxes:** Individuals who were wealthier and more educated showed higher levels of acceptance for increasing gasoline tax. Those who owned cars were less likely to accept than those who did not. **General preference for earmarking gasoline taxes for environmental purposes:** Preference for earmarking gasoline taxes was driven by an increase in the perceived effectiveness of taxes with earmarking, and because respondents did not trust government to redistribute revenue. **Increasing acceptance of revenue‐neutral gasoline taxes:** Support for revenue recycling increased when respondents were given monetary figures of how much income tax was reduced with a gasoline tax, rather than percentage reductions. People also preferred revenue recycling to decrease income taxes rather than sales taxes.
Jagers and Hammar (2009)	**Where and when:** Sweden, 2002, 2003, and 2004. **Type of policy intervention:** Existing carbon tax on transport fuels, with hypothetical increase in tax rate.	**Methodology:** Quantitative analysis of survey questionnaire. **Data collection:** Annual survey (repeated cross section) collected from sampling the National Registry by the SOM Institute at the University of Gothenburg (2002), authors (2003), and SOM Institute (2004). Over 1,000 responses from each year used for the analysis.	**Aversion to increasing carbon tax rates:** Swedes already see they have a high‐carbon tax rate, and would like increasing mitigation efforts to be met with alternative policies, including decreasing taxes on fuels that do not affect the climate, expanding public transport, and increasing information campaigns about traffic's contribution to climate change. **Potential acceptance of increasing carbon taxes with the right information devices:** Although Swedes have shown aversion to increasing carbon tax rates to support more ambitious climate mitigation, they are even more averse to increasing tax rates for income or municipal taxes. The implication is that if Swedes would like to increase mitigation efforts by decreasing taxes on low‐carbon fuels or expanding public transport, the Ministry of Finance would need to increase the rates of taxes that are even more unpopular than the carbon tax in order to finance the alternative mitigation options. Therefore, the authors argued, providing budgetary information on each mitigation proposal could increase support for increasing the carbon tax rate in contrast to alternative proposals. Providing data on the effectiveness of the existing carbon tax in decreasing emissions could also increase support. **Distribution of mitigation cost burden:** Respondents found it fairer to ask people who pollute the most to contribute a higher proportion of mitigation efforts, rather than each individual reducing the same proportion of emissions.
Bristow et al. (2010)	**Where and when:** Wales and southeast England, 2008. **Type of policy intervention:** Hypothetical carbon tax and personal carbon‐trading designs.	**Methodology:** Quantitative analysis of discrete choice experiment on personal carbon trading versus carbon tax, with attributes defining the design of each instrument (with differences in sectors covered, how revenues were recycled, and distribution of costs). **Data collection:** 79 respondents in Wales (recruited through a citizens' forum) and 208 respondents in southeast England (on‐street recruitment).	**Explanations of aversion to/acceptance of carbon taxes:** There was no clear indication of whether people preferred carbon pricing instruments in the form of a carbon tax or a personal carbon trading scheme. Preference was based on how the carbon pricing instrument was designed, based on the following factors: which emission sources were priced; how revenues were recycled; and the progressivity of the tax. **Use of carbon tax revenues:** Increased preference for carbon tax when revenue earmarked for environmental reasons.
Kallbekken and Aasen (2010)	**Where and when:** Norway, 2009. **Type of policy intervention:** Based on understanding of existing taxes on fuel, carbon, and electricity.	**Methodology:** Qualitative analysis of interview and focus group data. **Data collection:** Five focus groups, designed to reflect some variation in the demographic characteristics of Norway.	**Explanations of aversion to/acceptance of carbon/energy taxes:** People preferred subsidies over taxes in addressing environmental problems, as taxes represent a direct cost to the consumer. Participants also wanted government to provide more information on the scope of the environmental problem in order to build support for greater environmental action. **Use of carbon/energy tax revenues:** People had a strong preference for earmarking revenues from environmental taxes to address the original environmental problem, as it was seen as a way to improve the effectiveness of the tax, by reducing the cost of low‐carbon options (especially if participants expected a low elasticity of demand for the carbon‐intensive goods). Participants did not believe revenue‐neutral taxes were effective in reducing environmental impact, and did not understand the purpose of addressing social problems (like low unemployment) with revenues from an environmental tax (referred to as an issue‐linkage problem by Sælen & Kallbekken, 2011).
Kallbekken and Sælen (2011)	**Where and when:** Norway, 2010. **Type of policy intervention:** Alternative tax rates to existing fuel tax in Norway at the time of study.	**Methodology:** Quantitative analysis of survey questionnaire on acceptance levels for decreasing, keeping constant or increasing existing fuel tax rates, including removing the tax altogether. **Data collection:** Nationwide online survey of 1,177 Norwegians, representative of Norwegian public.	**Explanations of aversion to fuel taxes:** Findings showed that self‐interest in terms of personal cost from fuel tax was not a significant factor in people's aversion to fuel taxes. Instead, people's beliefs in the environmental effectiveness of the fuel tax in reducing emissions were significant. According to the authors, this finding suggests that communication strategies need to be used to show that people do respond to the fuel tax incentive by reducing consumption of transport fuels, which leads to decreasing emissions. Another reason why people are averse to fuel taxes is the fear that it disproportionately impacts low‐income households, or those who live in rural areas and are more dependent on driving as a form of transport. According to the authors, this finding suggests that the fuel tax can be designed to address these distributional concerns, through social cushioning measures targeted at low‐income households, or having differentiated fuel taxes between rural and urban areas. **Low tax rates preferred:** Voters on average preferred lower fuel taxes, which may also imply preference for reducing existing taxes.
Sælen and Kallbekken (2011)	**Where and when:** Norway, 2010. **Type of policy intervention:** Alternative tax designs to existing fuel tax in Norway at the time of study.	**Methodology:** Quantitative analysis of discrete choice experiment with design options differing in terms of tax rate and how revenues are recycled. **Data collection:** Nationwide online survey of 1,147 Norwegians, representative of Norwegian public.	**Use of fuel tax revenues:** Earmarking fuel taxes for environmental purposes increased acceptance of fuel tax to the majority of respondents, including increased acceptance of a hypothetical fuel tax increase of 15% above the official rate at the time of the study. The study showed that reasons for increased acceptance included people expecting to personally benefit from the use of earmarked revenues, and people perceiving earmarking for environmental purposes as a way to increase the effectiveness of the fuel tax, especially if they did not believe that the tax provides enough incentive to reduce emissions. Unlike other studies, the regression analysis shows that distrust in how governments distribute revenue is not among the reasons why Norwegians support earmarking revenues. Recycling fuel tax revenues to reduce income taxes did not achieve majority acceptance, as people could not understand the link between using revenue raised from addressing an environmental issue to be used to ameliorate a labor issue (showing the issue‐linkage problem). The least preferred option was transferring revenues to the general budget.
Brännlund and Persson (2012)	**Where and when:** Sweden, 2009. **Type of policy intervention:** Hypothetical climate policy instruments, including a hypothetical carbon tax.	**Methodology:** Quantitative analysis of discrete choice experiment of climate policy instruments with different resulting effects, including a carbon tax resulting in personal monthly cost ranging from 100 to 1,000 SEK. **Data collection:** Administered via online survey; responses from 2,400 respondents.	**Explanations of aversion to carbon taxes:** Carbon taxes that result in higher personal costs induced stronger aversion. **Preferred attributes of carbon tax:** The findings showed that people preferred climate policy instruments that support environmentally‐friendly technologies and have a progressive cost distribution. According to the authors, these findings support the idea of designing carbon taxes with these attributes.
Leiserowitz et al. (2013)	**Where and when:** USA, 2013. **Type of policy intervention:** Different types of carbon/energy taxes, shifts in fossil fuel subsidies, and regulations (based on existing and proposed policies in the USA).	**Methodology:** Quantitative analysis of survey questionnaire. **Data collection:** 830 respondents to national telephone survey in the USA.	**Explanations of aversion to/acceptance of carbon taxes:** There was majority support for low‐carbon research (72%), tax rebates for low‐carbon technologies (71%), regulating greenhouse gas emissions (67%), eliminating subsidies for the fossil fuel industry (59%), and requiring electric utilities to produce at least 20% of their electricity from renewable energy sources, even if it cost the average household an extra $100 a year (56% support). When evaluating the effectiveness of various global warming and energy policies, less than half of the sample were confident that: within the next decade, energy from solar and wind will be cheaper than energy from fossil fuels (48%); reducing the amount of oil the United States uses would protect from high gas prices (48%); subsidies are an effective way to support the diffusion of renewable energy (43%); a carbon tax is an effective way to support the diffusion of renewable energy (35%). **Use of carbon tax revenues:** Acceptance of revenue‐neutral energy taxes by reducing other taxes varied depending on the specific design: reducing the federal income tax (49% support); giving a tax refund to every American household (47%); reducing the federal payroll tax (45%). A straight carbon tax on fossil fuel‐producing or importing companies, if it cost US$180/year per average American household, was supported by 43% of the sample.
Gevrek and Uyduranoglu (2015)	**Where and when:** Turkey, 2012. **Type of policy intervention:** Hypothetical carbon tax.	**Methodology:** Quantitative analysis of discrete choice experiment that provides information on how different tax rates result in a range of personal monthly costs ranging from 2 to 6 Turkish Lira, and on how revenues are recycled. **Data collection:** Face‐to‐face interviews with 1,252 individuals in 16 Turkish cities.	**Explanations of acceptance of carbon taxes:** Respondents with high environmental awareness were more supportive of a carbon tax than those with low environmental awareness. **Tax rates:** Generally, respondents preferred a carbon tax with a lower tax rate. Respondents also preferred progressive tax rates to address distributional concerns related with the tax burden on low‐income households. Respondents with high environmental awareness and high income were more willing than others to accept a higher carbon tax rate. **Use of carbon tax revenues:** Respondents preferred to earmark carbon tax revenues to subsidize low‐carbon technologies, as it was perceived as a way to improve the effectiveness of the tax. Respondents preferred addressing distributional concerns through a progressive tax rate, rather than with targeted transfers (social cushioning) to low‐income households.
Alberini et al. (2016)	**Where and when:** Italy, 2014. **Type of policy intervention:** Climate policies, including a carbon tax, to reduce CO_2_ emissions from fossil fuels and renewable energy use in homes.	**Methodology:** Quantitative analysis of discrete choice experiment that provides different ranges of willingness to pay (WTP) per ton of CO_2_ reduction for each policy, with policies differing in attributes according to: (a) goal of policy (to improve energy efficiency or renewable generation); (b) specific policy, such as carbon tax, subsidies, standards, information‐based policies, and combinations thereof; (c) reduction of CO_2_ emissions of average household to baseline; and (d) cost of the policy to the respondent's household (on an annual basis). **Data collection**: Online survey of 1,005 respondents who own and reside in homes built in or before 2000.	**Explanations of aversion to carbon taxes:** Opposition was highest among those with lower education levels and those lacking awareness of climate change. **WTP to mitigate CO**_**2**_ **emissions:** WTP to mitigate 1tCO_2_e differs according to climate instrument. Carbon taxes had the lowest WTP at €6.44; the rate for information standards was €95.24; and for incentives (i.e., subsidies for renewables and energy efficiency), €133.15. (Note: WTP is the maximum amount an individual is willing to sacrifice to obtain a good or avoid something undesirable.)
Baranzini and Carattini (2017)	**Where and when:** Geneva, Switzerland, 2012. **Type of policy intervention:** Hypothetical carbon tax (with alternative label “climate contribution”).	**Methodology:** Initial qualitative interviews to inform survey design, followed by a face‐to‐face quantitative survey, split among those asked about a hypothetical carbon tax set at 120 CHF/tCO_2_, and those asked about a hypothetical “climate contribution” as an alternative label to a carbon tax. Quantitative analysis undertaken on survey questionnaire. **Data collection:** Initial interviews with 40 adults in Geneva, followed by survey of 338 respondents, who were randomly split with 158 being asked about a hypothetical carbon tax, and 180 being asked about a climate contribution.	**Introducing carbon taxes at low rates:** When respondents were asked to define the ideal tax rate, they tended to prefer a carbon tax rate that results in more moderate price increases on fuels than the default rate proposed by the survey. **Use of carbon tax revenues:** Where there was some distrust in government, carbon taxes tended to be more acceptable if revenue was earmarked for environmental purposes, in order to improve their perceived effectiveness (60% of respondents wanted earmarking for environmental purposes). This fits with the belief held by 52% of respondents, who did not believe carbon taxes to be effective. Social cushioning was the second most preferred option to recycling revenues, with a small minority preferring tax rebates to household and firms. **Communicating primary and ancillary benefits of carbon tax:** This is important as it increases the acceptability of the carbon tax, as the primary obstacle to the carbon tax was its perceived ineffectiveness, in reducing both global and local pollutants.
Ščasný, Zverinova, Czajkowski, Kyselá and Zagorsk (2016 )	**Where and when:** Czech Republic, Poland, and United Kingdom, 2015. **Type of policy intervention:** Targets for emission reductions for 2030 and 2050 (as set out in EU Climate and Energy Package).	**Methodology:** Quantitative analysis of discrete choice experiment containing four attributes of climate policy for EU mitigation efforts: emission reduction targets for each period year (as set out in 2014 Climate and Energy Package, with 40% reduction by 2030, and 80% reduction by 2050); and different options for sharing costs of mitigation. **Data collection:** Online questionnaires administered in each country; 4,098 responses.	**WTP for different EU climate targets for 2030 and 2050:** The United Kingdom had the highest WTP for meeting the 2020, 2030, and 2050 targets, followed by the Czech Republic. Both countries showed support for the 2014 Climate and Energy Package targets. The study shows in Poland there was a negative WTP, but it is not statistically significant. However, Polish respondents did prefer keeping the current targets, as stated in the 2020 targets. **Burden sharing rule among countries:** Respondents in the Czech Republic and the United Kingdom preferred the distribution of costs for reducing greenhouse gas emissions to be based on those who emit the most paying a higher cost (or in aggregate, emissions per country). Polish households were less willing to distribute burden sharing on emissions per country, and did not have a preference over the other types of burden‐sharing rules.
Carattini et al. (2017)	**Where and when:** Switzerland, 2015. **Type of policy intervention:** ETR on nonrenewable fuels (ballot) and hypothetical carbon tax.	**Methodology:** Two surveys, following the vote on a popular initiative suggesting to replace the existing value‐added tax with a tax on nonrenewable energy. In one survey, this specific design is compared with other alternative (hypothetical) designs, with different tax rates and use of revenues. Quantitative analysis of two sets of data: **“**VOX” opinion survey on voting behavior, and discrete choice experiment on alternative policy design. Discrete choice experiment respondents were previously contacted by mail with information about the survey and the different tax designs, whose effects on the economy, low‐income households, and greenhouse gas emissions had been simulated with a computable general equilibrium model of the Swiss economy. **Data collection:** Surveys administered after the referendum. VOX survey administered by telephone to 1,500 respondents and discrete choice experiment administered via telephone survey to 1,200 respondents.	**Explanations of aversion to ETR:** 92% of voters voted “no” in the 2015 referendum. The main reasons for this were concern that increased energy tax rates would have a disproportionate impact on low‐income households and firms vulnerable to global competition, and the perception that the nonrenewable energy tax would be ineffective. **Concern over tax rates:** The ballot survey suggested that most concerns were related to the high tax rate that would have been necessary (especially in the future) to completely replace the revenues from the value‐added tax. The discrete choice experiment provided additional evidence on the negative relationship between tax rate and acceptability. In this respect, people with low levels of climate change concern tended to have a higher sensitivity to tax rates, while people with stronger climate change concern tended to pay less attention to price levels. **Importance of providing full information, including credible modeled results, on the effects of different recycling options of energy tax revenues:** The VOX survey showed that people's acceptance of the tax on nonrenewable energy would have increased if revenues were earmarked for environmental purposes. However, the results from the discrete choice experiment arrived at a different conclusion, as that survey provided respondents with modeled impacts of each tax design proposal on: (a) the price of fuels, (b) greenhouse gas emissions, (c) purchasing power of the average Swiss household, and (d) purchasing power of the average low‐income household. By providing information on the comparative impacts between different recycling options, the discrete choice experiment reveals that information may change preferences for revenue recycling, as environmental earmarking is no longer the most popular option. That is, providing “full information,” including on the environmental and distributional effects of each type of recycling option, made more progressive forms of recycling (such as lump sum transfers or social cushioning measures) more acceptable, even more than earmarking for environmental purposes. The discrete choice experiment also shows that recycling revenues by reducing existing taxes was not popular (similar to the referendum results).
Kotchen, Turk, and Leiserowitz (2017)	**Where and when:** USA, 2016. **Type of policy intervention:** Carbon tax (hypothetical tax).	**Methodology:** Quantitative analysis of survey questionnaire. **Data collection:** Survey of 1,226 American adults drawn from GfK's Knowledge Panel, an online digital platform in which survey respondents are signed up as members for polled surveys. To seek national representativeness, the questionnaire was sent to members drawn using probability sampling methods, and key demographic variables were weighted, post survey, to match U.S. Census Bureau norms.	**Explanations of aversion to carbon taxes:** Respondents who believe global warming is currently happening were 35 percentage points more likely to support the carbon tax than those who stated they did not know if global warming is happening, while those who do not believe global warming is happening were 25 percentage points less likely to support the carbon tax, compared with those who did not know. Respondents' age, gender, years of education, and size of household they belong to, did not have a significant effect on the probability of supporting a carbon tax, but income and race did. For example, a US$10,000 increase in a household's annual income increased the likelihood of support by 1 percentage point. **WTP for carbon tax:** The average respondent household was willing to pay 14.4% more on their household energy bill in support of a carbon tax. In monetary terms, this translates to US$177 per year, with a confidence interval ranging from $101 to $587. However, there was a negative and statistically significant effect of cost: a $10 increase in the annual household cost of the tax reduced the probability of support by 1 percentage point. **Earmarking carbon tax revenues for specific purposes:** The most preferred option was to earmark tax revenue for developing clean energy (using 17.3% of carbon tax revenues), followed by funding improvements in infrastructure (using 14.5% of carbon tax revenues). Respondents also supported using carbon tax revenue to help communities—particularly low‐income communities most vulnerable to climate change—for assistance to adapt to climate change (using a total of 15% of revenues). More than 70% of respondents supported using 10.4% of carbon tax revenue to compensate workers in the coal mining industry, who could lose their jobs as a result of the carbon tax. The study calculates that earmarking this percentage of carbon tax revenue could lead to paying US$146,000 to all coal mining workers if the passage of the carbon tax was to lead to the entire industry shutting down. **Options of using carbon tax revenue:** The options which received over 50% support include reducing the national debt and federal income taxes (by using 12.7 and 9.9% use of carbon tax revenues, respectively). Those taxes that received less than 50% support to be reduced with the carbon tax revenues include the federal payroll taxes (e.g., social security and Medicare) and corporate taxes.

## APPENDIX B 1 EMPIRICAL FINDINGS TESTING FACTORS AFFECTING THE ACCEPTABILITY OF OTHER “PIGOVIAN” TAXES

**Table:**

Study (listed in order of publication year)	Location, year and type of policy intervention tested	Methodological details	Empirical findings
Odeck and Bråthen (2002)	**Where and when:** Norwegian cities of Bergen (tax introduced in 1986), Oslo (tax introduced in 1990), and Trondheim (tax introduced in 1992) **Type of policy intervention:** Road user congestion charging (existing).	**Methodology:** Quantitative analysis of survey questionnaire on road user attitudes, where respondents were asked to state, from a list of possible alternatives, their positive or negative attitudes toward the implementation of the road toll. **Data collection:** Annual road user attitude survey collected in Norway, with data for each city specific to the years before and after the congestion charge was introduced.	**Phasing in congestion charging:** Negative attitudes toward road toll charging declined a year after implementation (in comparison with a year before the introduction of the tax) in all three cities, with negative attitudes in Bergen and Trondheim decreasing to below 50%, and from 70 to 64% in Oslo. The study suggests that before introduction, people are less aware of the benefits of the toll and therefore only use anticipated costs to form their beliefs. In comparing how charges were introduced in each city, it was found that introducing taxes at a lower rate decreased negative attitudes. The study also highlights the importance of using information campaigns to show how charging may be the best policy option to address the original problem of road congestion.
Schade and Schlag (2003)	**Where and when:** Athens, Greece; Como, Italy; Dresden, Germany; and Oslo, Norway, 1998–1999. **Type of policy intervention:** Road user congestion charging (hypothetical for Athens, Como and Dresden at the time the study was administered, but existing for Oslo).	**Methodology:** Quantitative analysis of discrete choice experiment between two hypothetical policy packages which have “strong” and “weak” measures. These packages differ according to tax rates and how revenues are recycled. **Data collection:** Mailed surveys to motorists in each city (total sample size is 954 with 150 from Athens, 238 from Como, 281 from Dresden and 285 from Oslo).	**Explanations of aversion to/acceptance of congestion charging:** The strongest factor for accepting the charge was values held by peers/society (rather than personal beliefs) in addressing the problem of congestion through charging. The second strongest factor was expectations of how the charge would impact people's situation. The weak policy package, which had lower rates and used revenue to lower the costs of transport (rather than to decrease labor income as in the strong package), had greater acceptance in all cities. According to the authors, this suggests that there is greater acceptance of policy packages that introduce taxes at a lower rate, and use tax revenues to compensate affected constituents through other measures.
Halbheer, Niggli, and Schmutzler (2006)	**Where and when:** Switzerland, 1977–2003. **Type of policy intervention:** 45 Swiss referenda that have some relation to the environment: 24 on transport, 13 on energy, and 8 on landscape preservation and agriculture.	**Methodology:** Quantitative analysis of ballot outcomes. **Data collection:** Ballot data for the 45 referenda held between 1977 and 2003 in Switzerland.	**Explanations of aversion to/acceptance of environmental taxes:** Environmental referenda were not more likely to be rejected (or accepted) if they included an environmental tax. Policies that were most likely to be rejected were ones that restricted consumer choice (e.g., limiting the driving of cars). In fact, environmental referenda proposals which included taxes with a relatively low rate had higher acceptance rates than proposals that included strict regulations limiting consumer choice.
Gaunt et al. (2007)	**Where and when:** Edinburgh, UK, 2005. **Type of policy intervention:** Road‐user congestion charging (after proposal failed in referendum).	**Methodology:** Quantitative analysis on survey questionnaire. **Data collection:** Postal self‐completion questionnaire sent to 1,300 randomly selected households, with an approximately 25% response rate.	**Explanations of aversion to/acceptance of congestion charging:** Self‐interest was the main motivator in rejecting the scheme: opposition by car owners was greater than support from noncar owners, cyclists, and bus users. Although congestion was acknowledged as a problem, voters did not believe congestion charging would be effective in reducing congestion and improving environmental conditions (it was believed that car owners would pay the charge and still drive in congestion zones). **Opposition to phasing in congestion charging:** Respondents feared the introduction of congestion charging at a low rate would eventually lead to increasing rates. **Earmarking to address congestion not always accepted:** The promise of improving public transport was not believed by the public. Plus, responses indicated a belief that existing public transport was already good. Additionally, there was a lack of trust in government in how tax revenues would be spent.
Schuitema and Steg (2008)	**Where and when:** Netherlands, no date provided. **Type of policy intervention:** Road‐user congestion charging (hypothetical).	**Methodology:** Quantitative analysis on survey questionnaire. **Data collection:** 507 Dutch respondents, drawn from a Dutch marketing firm's database. Respondents were randomly selected from a database of commuters who experienced congestion during the morning rush hour at least twice a week.	**Use of congestion charging revenues:** Acceptability of congestion charging depended on how revenue was recycled—especially if those taxed felt they were compensated for the personal costs of congestion charging. There was increased acceptance if congestion charging was to be used to reduce other car‐related taxes. Findings also show the importance of information campaigns to show how congestion charging will create benefits through revenue recycling.
Winslott‐Hiselius et al. (2009)	**Where and when:** Stockholm, Sweden, 2004–2006. **Type of policy intervention:** Road‐user congestion charging made permanent after a trial period conducted in the first half of 2006.	**Methodology:** Quantitative analysis of survey questionnaire. **Data collection:** Total of 1,600 telephone interviews conducted during 2004, 2005, and 2006.	**Phasing in congestion charging:** 15% of respondents were more positive about the congestion charge during the trial than before it started. This increase seems to be enough to make congestion charging acceptable, as 51.3% of the inhabitants in the city of Stockholm voted in favor of a permanent solution with congestion charges after the trial period. **Benefits of trial period:** Respondents who increased their acceptance of congestion charging during the trial period personally experienced the benefits of congestion charging (in terms of reduced congestion and improved air quality), in contrast to their perception before the trial period. **Importance of improving public transport:** The government committed to improving public transport by running more services during the trial period, which caused some road users to accept using public transport in place of their cars, and at the same time avoided complaints about overcrowding on public transport from commuters.
Schuitema et al. (2010)	**Where and when:** Stockholm, Sweden, 2005 and 2006. **Type of policy intervention:** Road‐user congestion charging made permanent after a trial period conducted in the first half of 2006.	**Methodology:** Quantitative analysis of survey questionnaire. **Data collection:** Mailed survey of 143 respondents interviewed in December 2005 and August 2006 (i.e., before and after trial period).	**Greater acceptance of congestion charging after trial period than before:** The reason for increased acceptance after the trial period was that people were able to experience the benefits of congestion charging (e.g., reduced congestion, parking problems, and pollution) during the trial period, and therefore saw the effectiveness of congestion charging (especially if it led to individuals reducing own car use by having alternative options available at the same time). Furthermore the costs of congestion charging were not as high as participants expected before the trial period.
Kallbekken et al. (2011)	**Where and when:** Innsbruck, Austria. **Type of policy intervention:** Hypothetical Pigovian tax schemes.	**Methodology:** Quantitative analysis of data generated from a lab experiment with a market and an externality. The lab experiment consisted of a market for a fictitious good in which some buyers imposed external costs on others through their purchases. After initial trading periods without taxation, buyers participated in four votes, in which they faced binomial choices between the instrument being referred to as a tax or a fee, and with different rules for how to distribute the collected tax revenues (including a no tax scheme). After trading and voting finishes, subjects were given a questionnaire, to test their understanding of implications of tax schemes for additional costs to others, payoffs to themselves, and the group as a whole. **Data collection:** Experiment was conducted at the University of Innsbruck with a total of 160 students as participants.	**Tax aversion may be such that people vote against tax schemes that serve their own material self‐interest, while increasing social welfare:** Efficiency‐enhancing Pigovian taxes can increase individuals' pay‐offs as well as social (group) welfare. Interestingly, in this lab setting, providing information on how Pigovian taxes work seemed not to reduce tax aversion. **Labeling and use of Pigovian tax revenues:** Respondents preferred tax schemes that earmarked the revenue to target the original externality problem. When taxes were earmarked, it did not matter if the instrument was labeled a “tax” or a “fee.” However, if tax revenue was redistributed through lump‐sum transfers, then the label “tax” did reduce support in comparison to “fee.”
Cherry et al. (2012)	**Where and when:** Colorado, USA, 2011. **Type of policy intervention:** Pigovian taxes, subsidies, and regulations.	**Methodology:** Quantitative analysis of data generated from a lab experiment with a market and an externality. The lab experiment consisted of a market for a fictitious good in which some buyers imposed external costs on others through their purchases. The experiment had three treatment variables, which altered the characteristics of the policy that participants could support, in a referendum, to address the externality: instrument type (tax, subsidy, and regulation), efficiency (full measure, half measure, and no policy), and language (label and generic; see next column). **Data collection:** Lab experiment taking place at Colorado State University, involving 95 subjects participating in five sessions, each session consisting of nine referenda.	**Explanations of aversion to/acceptance of carbon taxes:** Although people were strongly averse to taxes, this finding is not specific to taxes only, as people generally were averse to any type of market intervention. However, they preferred subsidies over taxes and taxes over regulations that limit consumption levels. **Preferred tax rates:** In the case of regulation, “half” measures were preferred to more efficient “full” measures. In the case of carbon taxes (and subsidies), “full” measures were preferred when contrasted against “half” measures, but not necessarily when contrasted against the status quo (no policy).
de Groot and Schuitema (2012)	**Where and when:** Bournemouth, UK, 2010. **Type of policy intervention:** Interventions to address car use and littering.	**Methodology and data collection:** Quantitative analysis of a discrete choice experiment which had participants choose between policy interventions to address two different environmental problems—car use and littering. The issues were to be addressed with a tax/fine or subsidized low‐carbon options. Participants were also given information on the level of support for each intervention among the UK population. **Data collection:** Responses from 123 individuals recruited from public spaces in Bournemouth.	**Explanations of aversion to/acceptance of different policy interventions:** Policies that subsidized low‐carbon options were more accepted than policies that imposed a direct cost on polluters, as the latter were seen to be more coercive in restricting polluting behavior. Policies that targeted perceived “high cost” behaviors (such as reducing car use) were less acceptable than policies that targeted behaviors that had a low perceived cost to change (e.g., reducing littering). **People were more willing to support policies that other people also support:** People were willing to accept policies that they initially rejected (e.g., increasing taxes on car use) if they saw that their peers/the general population was willing to support them. According to the authors, this finding demonstrates the importance of governments taking long‐term action to build support for protecting the environment (including for environmental taxes). It also shows that when there is high support for environmental taxes, governments should disclose these statistics to show undecided voters that these taxes are widely supported.
Cherry et al. (2014)	**Where and when:** Copenhagen, Denmark, 2009. **Type of policy intervention:** Hypothetical Pigovian tax.	**Methodology:** Quantitative analysis of data generated from a lab experiment with a market and an externality. The experiment focused on Pigovian taxes to address the externality and had a two‐by‐two design: (a) the rates are set at threshold level or at the full tax and (b) Pigovian tax is preceded with a trial run or not. **Data collection:** Lab experiment held at the University of Copenhagen, which had nine sessions that in total involved 170 students.	**Phasing in Pigovian taxes:** Trial runs increased acceptability for Pigovian taxes when participants observed benefits during the trial, suggesting that people's aversion to Pigovian taxes was due to their misperception about the purpose and effects of the tax. Trial runs increased acceptance of taxes set at the threshold rate (where tax is imposed only after a minimum level of consumption) and also at the full rate (taxes imposed on all consumption). The experiment shows trial periods reduced aversion because people were able to perceive benefits of the tax. Still, it should be noted that the preference for the threshold tax was greater than for the full tax.
Heres et al. (2015)	**Where and when:** Bilbao, Spain, 2012. **Type of policy intervention:** Hypothetical subsidies versus hypothetical Pigovian taxes.	**Methodology:** Quantitative analysis of experimental data obtained in a lab experiment, where participants faced the same economic incentives but had different information on them. **Data collection:** Eight experimental sessions (involving four stages) at the University of Bilbao, involving a total of 195 participants anonymously interacting in groups of five via computer terminals.	**Explanations of aversion to/acceptance of Pigovian taxes:** When there was a lack of budgetary information provided for either instrument, people were more likely to prefer a subsidy over a Pigovian tax, as they expected to obtain a higher personal payoff with a subsidy. However, subjects did not expect a subsidy or the tax to differ in their effectiveness in reducing negative externalities. **Importance of providing complete budgetary information on subsidies and Pigovian taxes to help voters choose from different instruments:** Findings showed increased acceptance of using either subsidies or Pigovian taxes to address negative externalities when more (or complete) budgetary information involving either instrument was provided (for subsidies the information involved how it would be financed, while for taxes it was how the revenues would be used). In fact, providing complete budgetary information on taxes changed participants' perception that taxes only impose costs on consumption, and demonstrated how people can gain from taxes through distribution of tax revenues. When information was incomplete, the results suggested that subsidies were expected to increase individual payoffs by a larger amount than redistribution of tax revenue would achieve. This finding supports the idea that voters should receive complete budgetary implications for all subsidies and taxes in order to judge which instrument would benefit them the most.
Tiezzi and Xiao (2016)	**Where and when:** Pittsburgh, USA. **Type of policy intervention:** Pigovian tax (hypothetical).	**Methodology:** Quantitative analysis of lab experiment data. Lab experiment is designed with market that has a two‐by‐two treatment design: (a) either the external costs of consuming a fictitious good occur in the same time period as when the good was consumed (“No Delay” treatment), or the external costs occur in a later time period to when the good was consumed (“Delay” treatment); and simultaneously (b) either the group started without a revenue‐neutral Pigovian tax (in which case, groups voted on whether to introduce a revenue‐neutral Pigovian tax), or with a revenue‐neutral Pigovian tax (where the participants voted on whether to remove the tax). **Data collection:** Conducted at the Pittsburgh Experimental Economics Laboratory (PEEL) with 12 sessions involving a total of 212 student participants.	**Explanations of aversion to Pigovian taxes:** When people did not immediately experience the effects of a negative externality at the time of consumption, they were less willing to accept Pigovian taxes to change consumption behavior, and preferred to delay implementing a tax. This unwillingness to accept the Pigovian tax occurred even when the tax was framed as the default policy option for addressing the externality problem. Since, according to the authors, a reasonable discount rate does not suffice to explain this pattern, it is suggested that voter aversion to Pigovian taxes is driven by the complexity of the underlying externality, in this case represented by the delayed response of the externality to the change in pollution levels. **Aversion to Pigovian taxes declined after participants had become aware of the benefits of immediately implementing taxes to reduce the costs of the externality in future periods:** The majority of respondents who voted against the tax switched views when they felt the immediate benefits of the tax in reducing the problems of the externality. According to the authors, this finding is worrying as the negative effects of externalities such as climate change are not felt in the same time period as when polluting activities occur (i.e., there is a delayed negative effect). However, when explicit information about the intertemporal trade‐off (between consuming now and bearing costs later) was provided, participants were more willing to accept the tax. **Suggestions to introduce Pigovian taxes:** According to the authors, the implications of the study are that trial periods for Pigovian taxes are more easily accepted when the benefits can immediately be perceived. In cases where the benefits of the tax are not immediately experienced, the study suggests having government campaigns that can explain the costs of delaying action to help voters accept tax in earlier time periods.
Cherry et al. (2017)	**Where and when:** Colorado, USA. **Type of policy intervention:** Pigovian tax, subsidy, and regulations limiting consumption.	**Methodology:** Quantitative analysis of lab experiment data. The lab experiment has an experimental market consisting of five buyers who buy a fictitious good that imposes external costs on others. The experiment provides six policy options that vary across instrument type (tax, subsidy or quantity regulation) and efficiency level (full and half). After the lab session, a questionnaire is given to participants to elicit their world views. **Data collection:** Eight sessions involving 160 students from Colorado State University.	**Explanations of aversion to Pigovian taxes:** Generally, respondents were averse to any type of policy intervention to correct for negative externalities. Subsidies were the most preferred policy intervention in comparison to taxes and quantity restricting regulations (quotas). However, world views do play a role in the level of aversion to policy interventions, and the type of policy intervention. The study found that people who were more hierarchical and/or individualistic were more averse to policy intervention than those who were more egalitarian and/or communitarian. “Coercive” instruments were more offensive to individualists, but instruments that enable redistribution were more attractive to egalitarian types. **Introducing Pigovian taxes:** The study found that in the initial absence of corrective policies for externalities, people preferred starting at a half rate than full rate for Pigovian pricing instruments. It should be noted that world views (hierarchical vs. egalitarian, and individualistic vs. communitarian) had no significant effect on preference over the rate at which policies should be set. **Experience does not increase acceptance for Pigovian taxes:** Aversion to policies declined for subsidies and quantity restricting policies if participants had experienced these instruments in previous periods. This decline in aversion increased the likelihood of support in current referenda. However, in this study, this effect was not found for Pigovian taxes.
Carattini, Baranzini, and Lalive (2018)	**Where and when:** Canton of Vaud, Switzerland, 2012 and 2013. **Type of policy intervention:** Pricing household waste by the bag (unit pricing).	**Methodology:** Econometric analysis (difference‐in‐difference approach) that compares acceptance and effectiveness of pricing waste by the bag. The analysis exploits the decision by the Federal Supreme Court of Switzerland to mandate the implementation of unit pricing in all municipalities in the canton. Municipalities implementing the policy represented the treatment group. Municipalities that already had unit pricing prior to the Supreme Court decision represented the control group. **Data collection:** Telephone interviews with households in municipalities in the “control” group (48 municipalities) and “treatment” group (22 municipalities), with a total of 193 households participating. Interviews were realized both before and after the treatment occurs. Administrative data from all municipalities were used to measure per capita household waste (from 2008 to 2013). Interviews with 44 municipalities on policies to help individuals dispose of their waste were also carried out.	**Waste tax does change behavior:** Pricing household waste by the bag was shown to decrease waste by 40%, and to increase recycling of aluminum and organic waste. **Phasing in waste taxes:** People's perceptions toward pricing waste by the bag improved significantly once they experienced the policy. A substantial proportion of respondents revised their beliefs concerning the policy's effectiveness and fairness: respondents were willing to support a price 70% higher for a bag of waste after the policy implementation than before.
